# Global Landscape of Native Protein Complexes in *Synechocystis* sp. PCC 6803

**DOI:** 10.1016/j.gpb.2020.06.020

**Published:** 2021-02-24

**Authors:** Chen Xu, Bing Wang, Lin Yang, Lucas Zhongming Hu, Lanxing Yi, Yaxuan Wang, Shenglan Chen, Andrew Emili, Cuihong Wan

**Affiliations:** 1 School of Life Sciences and Hubei Key Laboratory of Genetic Regulation and Integrative Biology, Central China Normal University, Wuhan 430079, China; 2 Donnelly Centre for Cellular and Biomolecular Research, University of Toronto, Toronto, ON M5S 2E8, Canada; 3 Departments of Biochemistry and Biology, Boston University, Boston, MA 02215, USA

**Keywords:** Protein–protein interaction, Cyanobacterium, Co-fractionation, Photosynthesis, Function prediction

## Abstract

*Synechocystis* sp. PCC 6803 (hereafter: *Synechocystis*) is a model organism for studying **photosynthesis**, energy metabolism, and environmental stress. Although known as the first fully sequenced phototrophic organism, *Synechocystis* still has almost half of its proteome without functional annotations. In this study, by using **co-fractionation** coupled with liquid chromatography-tandem mass spectrometry (LC-MS/MS), we define 291 multi-protein complexes, encompassing 24,092 **protein–protein interactions** (PPIs) among 2062 distinct gene products. This information not only reveals the roles of photosynthesis in metabolism, cell motility, DNA repair, cell division, and other physiological processes, but also shows how protein functions vary from bacteria to higher plants due to changes in interaction partners. It also allows us to uncover the functions of hypothetical proteins, such as Sll0445, Sll0446, and Sll0447 involved in photosynthesis and cell motility, and Sll1334 involved in regulation of fatty acid biogenesis. Here we present the most extensive PPI data for *Synechocystis* so far, which provide critical insights into fundamental molecular mechanisms in cyanobacteria.

## Introduction

Cyanobacteria represent the phylogenetic ancestors of chloroplasts from present-day plants [1], [2]. The oxygen generated by oxygenic photosynthesis is believed to change the atmospheric composition and promote biodiversity on earth [3]. *Synechocystis* is a unicellular photoautotrophic cyanobacterium, which is an ideal model organism for studying photosynthesis, energy metabolism, and environmental stress [4], [5]. The genome of *Synechocystis* was sequenced in 1996 [6], and its proteome has also been well analyzed in the last two decades [7], [8]. However, about two-thirds of its proteins in the UniProt database are listed as “hypothetical”, and most of them lack functional annotation.

Completion of many important biological functions relies on stable physical interactions between two or more proteins. Protein–protein interactions (PPIs) are critical to understand the fundamental molecular biology of organisms, which can be used for predicting annotation, finding new drug targets, and so on [9]. However, information on *Synechocystis*’s PPIs is quite a deficiency. Researchers have tried to analyze PPIs by using yeast two-hybrid (Y2H) assays and several kinds of prediction algorithms [10], [11], [12], [13]. However, in the STRING database, only 6510 PPIs involving 1876 proteins in *Synechocystis* were annotated with “experiments” until January 2019, which contained PPIs with relevant information transferred from other organisms. Even for those well-known *Synechocystis* protein complexes, for example, the photosystem II (PSII) assembly, putative assembly factors remain to be identified to fully understand the biogenesis process [14], [15]. The phototactic movement of cells is influenced by the motility apparatus and light, but the link between photoreceptors and the motility apparatus remains uncertain [16], [17]. Thus, globally mapping the PPI connectivity network of *Synechocystis* can provide a useful resource for functional inference.

High-throughput methods have been applied to systematically determine global protein interaction maps in many organisms, such as *Escherichia coli*, fly, worm, yeast, and human [18], [19], [20], [21]. Several techniques have been developed to identify protein complexes at the proteome scale, *e.g.*, Y2H, affinity purification mass spectrometry (APMS), and co-fractionation coupled with mass spectrometry (CoFrac-MS). Among these methods, CoFrac-MS can rapidly detect hundreds of endogenous macromolecular complexes composed of multiple stably-associated proteins under near native physiological conditions [22]. CoFrac-MS has been broadly used to identify PPIs at the proteome scale [23], [24], [25].

To reveal PPIs and uncover novel biological functions, we applied CoFrac-MS to analyze the protein complexes of *Synechocystis*. In this work, we predict the membership of 291 protein complexes containing 24,092 highly confident PPIs among 2062 proteins. This network facilitates our comprehensive understanding of the relationship between photosynthesis and other functions, such as carbohydrate metabolic process, signal transduction, ion transport, cell division, and transcription*.* In addition, we applied the PPI information to predict and confirm the new functions of proteins, such as Sll0445, Sll0446, Sll0447, and Sll1334. This work allows us to comprehensively understand the fundamental molecular organizations and mechanisms of *Synechocystis* and other cyanobacterial species.

## Results and discussion

### Workflow for protein complex identification in *Synechocystis*

**Figure 1:**
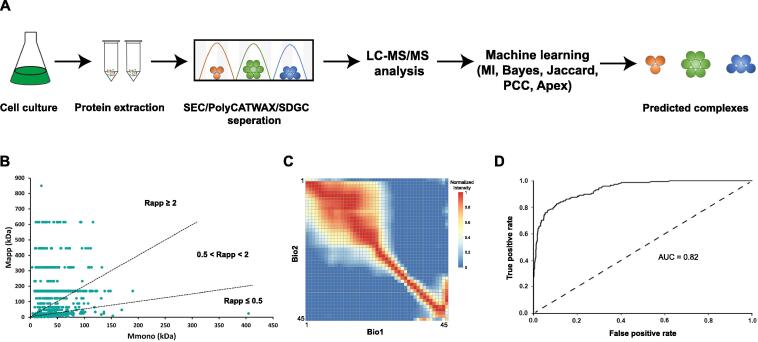
**Workflow for the identification of native protein complexes** **A.** Schematic diagram of co-fractionation, LC-MS/MS, and machine learning. Lysates containing a mixture of protein complexes were produced, and then separated by SEC, IEX, or SDGC. Proteins in each fraction were digested with trypsin and analyzed using nano-LC-MS/MS. Putative PPIs were predicted by machine learning using EPIC toolkits. **B.** The calculation of Rapp, which can accurately reflect the oligomerization state of proteins. A protein with Rapp ≥ 2 means that it has interactions with other proteins. **C.** Heatmap of the Pearson correlation coefficients of the protein quantification signals in two SEC biological replicates. **D.** Receiver operating characteristic curve of machine learning. SEC, size-exclusion chromatography; IEX, ion-exchange chromatography; SDGC, sucrose density gradient centrifugation; LC-MS/MS, liquid chromatography-tandem mass spectrometry; PPI, protein–protein interaction; EPIC, Elution Profile-based Inference of Complexes; Rapp, the ratio of Mapp to Mmono; Mapp, apparent molecular mass; Mmono, predicted molecular mass of the monomer; AUC, area under the curve.

The experimental workflow is similar to previous work (Figure 1A) [20]. Total protein mixtures were extracted from *Synechocystis*, separated by ion-exchange chromatography (IEX), size-exclusion chromatography (SEC), or sucrose density gradient centrifugation (SDGC), and analyzed by LC-MS/MS. SEC is advantageous for distribution of protein according to different molecular weight (MW) [24]. However, SEC is not sufficient to resolve protein complexes with MW beyond its valid separation range. To improve separation efficiency, we applied IEX as an additional separation technique. These two techniques are complementary in which IEX can resolve protein complexes that are not distinguishable during SEC (Figure S1).

In total, 181 fractions were collected, and 2906 proteins were confidently identified (Table S1). Proteins were separated effectively according to their MW and isoelectric point (pI) (Figure S2). The value of Rapp, the ratio of Mapp (apparent molecular mass, Figure S3) to Mmono (predicted molecular mass of the monomer), was evaluated to estimate whether a protein was involved in a stable heteromeric complexes on the SEC column [26], [27]. Rapp ≥ 2 was used to classify proteins predicted to be within a complex, while Rapp ≤ 0.5 suggested that protein degradation occurred during the protein extraction process (Figure 1B). The reproducibility between biological replicates was confirmed by Pearson correlation coefficient analysis of the profiles of spectral counts recorded for each identified protein (Figure 1C).

The components of stable protein assemblies co-elute together and can be detected by CoFrac-MS, while unstable assemblies become dispersed and so present unsatisfactory correlation profiling [19]. For example, photosystem components, NAD(P)H-quinone oxidoreductase, RubisCO complexes, and C-phycocyanin, tend to have a consistent correlation profiling (Figure S4). These protein elution profiles in turn confirm that CoFrac-MS is a powerful tool to explore global protein interactions in organisms. We confirm the final set of PPIs by machine learning using Elution Profile-based Inference of Complexes (EPIC) [28], with the classifier trained based on 47 reference ‘gold standard’ macromeocules annotated in the UniProt and IntAct databases (Table S2). The output of the prediction procedure was used to predict the components of multi-protein complexes (Figure 1D, Figure S5).

**Figure 2:**
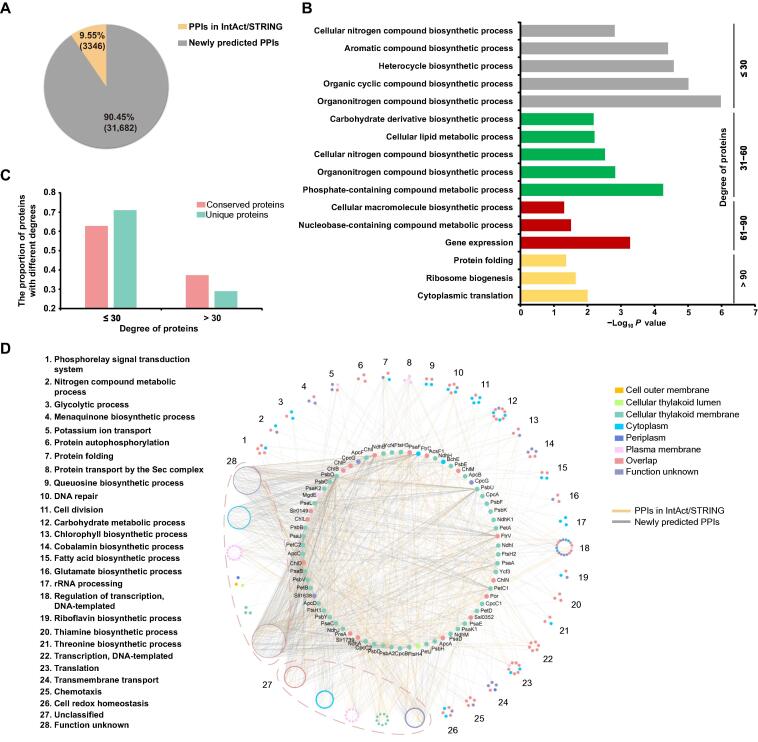
**The characteristics of predicted PPIs** **A.** Pie chart showing the overlap of predicted co-complexed PPIs with PPIs from IntAct and STRING databases. **B.** GO analysis of proteins with different degrees. Degree means the number of edges that one protein links to other proteins in the network (*P* < 0.05). **C.** The degree distributions of conserved proteins and unique proteins in *Synechocystis.* The conserved protein refers to the protein showing homology with proteins in *E. coli*, *A. thaliana*, *H. sapiens*, and *S. cerevisiae.***D.** The photosynthesis-associated proteins classified based on their functions. Each cluster has three or more proteins aggregated with the same GO term. Node colors represent the subcellular localizations of proteins. “Overlap” means that the proteins have multiple subcellular localization annotations. GO, Gene Ontology.

From this analysis, 2214 proteins were predicted to participate in 35,028 highly confident pairwise associations (Table S3). Our study provides the most extensive physical interaction network for *Synechocystis* to date, with ∼ 10% of the putative PPIs overlapping with previously reported pairs in a curation database, such as STRING and IntAct (Figure 2A). We compared the distribution of interactions/degrees, which reflects the critical connectivity of each protein in the network [29] we found with that reported for several different model organisms, such as *E. coli*, *Saccharomyces cerevisiae*, *Arabidopsis thaliana*, and *Homo sapiens*, according to the mentha database [30]. We observed that most of the proteins in *Synechocystis* tend to have low degrees, and that interacting proteins tend to be annotated to different metabolism pathways in *Synechocystis*, such as organonitrogen, aromatic, and cellular nitrogen compound biosynthetic process (Figure 2B, Figure S6). In contrast, ribosomes and heat shock proteins present high degrees in our dataset and are conserved across different species (Figure 2B); for example, DnaK1 and DnaJ occupy important positions in metabolism [31]. Other conserved proteins tend to have high degrees [32]. Additionally, proteins that have homologs in *E. coli*, *A. thaliana*, *H. sapiens*, or *S. cerevisiae* were found to have higher degrees than proteins unique to *Synechocystis* (Figure 2C).

### Photosynthetic apparatus involved in multiple metabolic pathways

As a model organism to study photosynthesis, *Synechocystis* has a classical photosystem structure containing photosystem I (PSI), PSII, cytochrome b_6_f complexes, and ATP synthase complexes [5]. There are 70 proteins annotated with photosynthesis, and 760 proteins were found to have direct interactions with these proteins in our dataset (Table S4). Photosynthesis-associated proteins were clustered into 26 different groups based on their functions, including phosphorelay signal transduction system, potassium ion transport, DNA repair, cell division, carbohydrate metabolic process, transcription, DNA-templated, translation, chemotaxis, and cell redox homeostasis (Figure 2D). The results indicate how photosynthesis influences many critical biological processes other than photosynthetic carbon fixation. For example, we observed that several CheA-like proteins interact with photosynthetic core proteins in our database. These CheA-like proteins have phosphorelay sensor kinase activities and participate in cellular chemotaxis [16]. These observations suggest that cells might control motility direction by regulating the state of CheA-like proteins through phosphorelay in photosynthesis.

Protein transport Sec complexes also had an association with photosynthetic proteins [14]. The subunits SecD and SecF might participate in the PSII assembly process by interacting with different PSII core proteins with a similar function as SecY (Figure 2D). SecY is the main transmembrane subunit of preprotein translocase that is essential for PSII assembly by interacting with YidC insertase to facilitate co-translational pD1 insertion [33], [34]. In our dataset, ChlD, a chlorophyll biosynthesis protein, was also observed to interact with SecD, indicating that it might be functional to deliver chlorophyll to the newly synthesized pD1, playing a similar role as ChlG (Figure 2D) [34]. Besides, although we observed that most proteins interacting with photosynthetic proteins are mainly localized in the cytoplasm, these proteins have possible roles in mobility or secretion according to localization identification results [35]. These results provide a clue for understanding how photosynthetic proteins may transmit signals and influence the physiological metabolism of cells.

### The landscape of native protein complexes in *Synechocystis*

**Figure 3:**
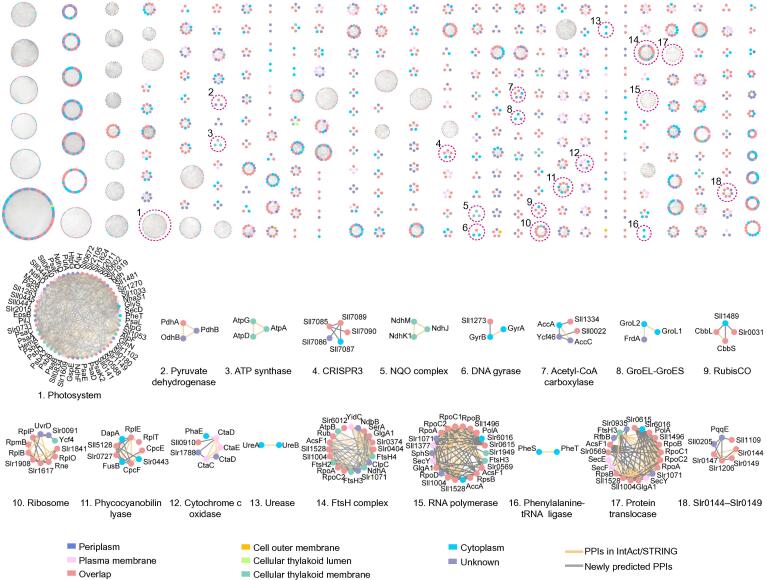
**Protein complex map of *Synechocystis*** The top part shows the global landscape of 291 *Synechocystis* protein complexes. The protein nodes are colored according to their subcellular localizations. “Overlap” indicates that the complex components have multiple subcellular localization annotations. The bottom part presents some of the known protein complex clusters, such as photosystem, ATP synthase, and ribosome, which are annotated with their name or abbreviation. Yellow lines between proteins indicate the interactions found in public databases, while gray lines indicate the interactions only reported in this study.

From the highly confident protein pair assignments from machine learning, we predicted 291 protein complexes with 24,092 PPIs using the ClusterONE clustering algorithm [36], which is implemented in the EPIC software. The set of predicted putative complexes contains well-known and highly-conserved complexes, such as PSI, RubisCO, and NADH dehydrogenase (Figure 3; Table S5). The functions of predicted complexes involved in photosynthesis, carbon absorption, nitrogen fixing, and electron transfer were also deduced. Besides, we also observed protein assemblies with unclear molecular function annotations, such as clustered regularly interspaced short palindromic repeats (CRISPR) system proteins Sll7085–Sll7090, photosystem proteins Sll0144–Sll0149, and ATP-dependent zinc metalloprotease FtsH complexes (Figure 3).

According to the computational analysis, *Synechocystis* has three types of CRISPR/CRISPR-associated (Cas) systems, but their exact functions remain unclear [37], [38], [39]. In our dataset, we found that some CRISPR3 proteins can form stable complexes, while protein interactions between different CRISPR systems were also observed (Figure S7). These results suggest that different types of CRISPR scanning systems coordinate with each other to defend cells from virus and plasmid invaders rather than functioning independently.

The hypothetical proteins Slr0144–Slr0149 are located on the thylakoid membrane and contain putative bilin binding domains, 4-vinyl reductase (V4R) domains, and 2Fe-2S cluster binding domains. They are mainly involved in photosynthetic repair [40]. The interactions of Slr0144–Slr0149 were independently validated by APMS (Figure S8).

Among the novel predicted complexes, we found that hypothetical proteins, Sll0445, Sll0446, and Sll0447 formed a stable association with pilus assembly proteins, Slr2015 and Slr2018, as well as with photosystem complexes. The physical interactions between Sll0445 and photosynthetic proteins were verified by APMS experiments (Table S6); APMS revealed additional candidate partners, presumably because different methods tend to catch different physical interactions [41]. It is worth mentioning that Slr2015 and Slr2018 interacted with Sll0445 by combining the results from co-fractionation and APMS (Figure S8). Slr2018 is located at the plasma membrane and is known to be regulated by SYCRP1, which is a cAMP receptor that influences cell motility in *Synechocystis* [42]. While Slr2018 shows no homology with other proteins, its cognate gene is adjacent to genes encoding Slr2015, Slr2016, and Slr2017, which have roles in pilus morphology and motility. These proteins were predicted to form protein complexes with photosynthetic proteins (Figure 3) [43], our results reveal a close connection presents between photosynthesis and cell motility in *Synechocystis*.

### Conserved complexes recruit different protein members across species

Half of our predicted protein complexes have conserved homologs in other species, like *A. thaliana* or *E. coli*, while the rest are unique to *Synechocystis* (Figure S9). The conservation of protein complexes was evaluated based on the number of proteins with known homologs in each complex. On average, the predicted protein complexes in *Synechocystis* have a higher similarity with plants (*e.g.*, *A. thaliana*) and bacteria (*e.g.*, *E. coli*) as compared to eukaryotes (*e.g.*, *H. sapiens* and *S. cerevisiae*), presumably reflecting overlap with the metabolic feature of Gram-negative bacteria and the photosynthetic apparatus of green plants. There are 920 proteins and 813 proteins with homologs in *E. coli* and *A. thaliana*, respectively (Table S7).

**Figure 4:**
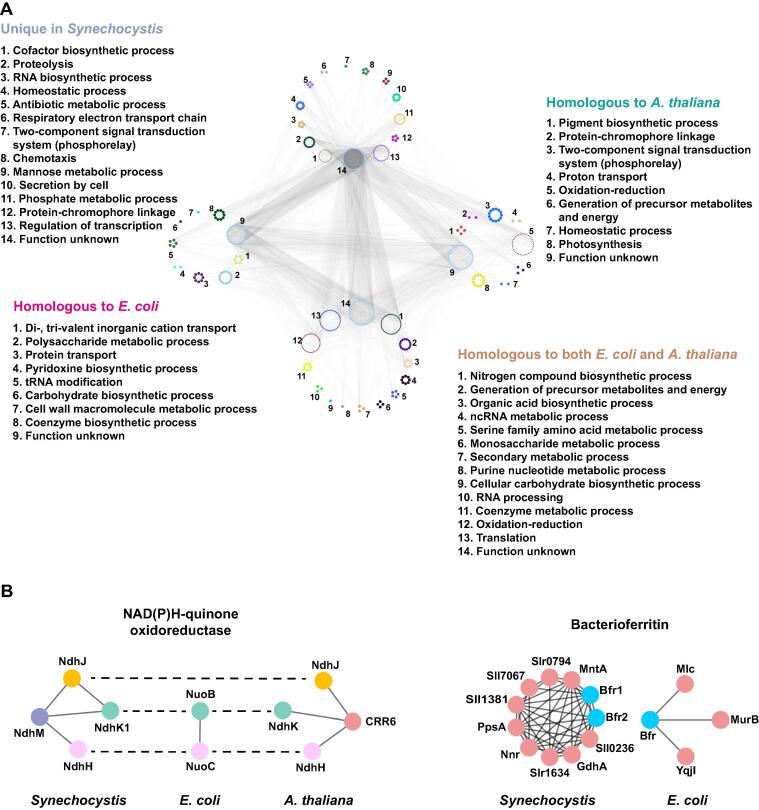
**Properties of evolutionarily conserved protein complexes** **A.** Proteins with homologs in either *E. coli* or *A. thaliana*, in both *E. coli* and *A. thaliana*, or unique in *Synechocystis* were grouped according to their representative biological processes*.***B.** Examples of PPI variations of NAD(P)H-quinone oxidoreductase (left) and bacterioferritin (right) across species. Physical interactions in *E. coli* and *A. thaliana* were collected from the mentha database. InParanoid orthologs of two or three species are depicted with the same colors. Proteins without homologs in other species are shown in red.

To examine the evolutionary relationship of *Synechocystis* assemblies with those of *A. thaliana* and *E. coli*, we separated our predicted complexes according to homology*.* The components of the predicted complexes were divided into four parts: unique in *Synechocystis*, homologous to *E. coli*, homologous to *A. thaliana*, and homologous to both *E. coli* and *A. thaliana* (Figure 4A). Proteins having homologs in both *E. coli* and *A. thaliana* retained functions in energy metabolism, organic synthesis, protein expression, and cellular regulation, which are considered as basic activities conserved across most species. Proteins with homologs in *A. thaliana* were mainly related to photosynthesis and pigment biosynthesis, which is consistent with the fact that one of the most remarkable features shared between *Synechocystis* and *A. thaliana* is photosynthetic capacity. Proteins with homologs in *E. coli* were primarily associated with the polysaccharide production, protein transport, and coenzyme biosynthesis, reflecting the similarities of *Synechocystis* and *E. coli* in terms of cellular structure and physiological properties [44], [45].

Notably, the terms of oxidation–reduction, homeostatic process, and two-component signal transduction system (phosphorelay) were assigned to more than one of these four groups. However, proteins that performed these functions and their interaction partners were different (Figure 4A). For example, proteins annotated as “oxidation–reduction” that have homologs in both *A. thaliana* and *E. coli* mainly participate in the “generation of precursor metabolites and energy” and “cellular respiration” processes, while proteins annotated as “oxidation–reduction” that have homologs only in *A. thaliana* are mainly associated with photosynthesis (Figure S10).

Here, we identified 14 NAD(P)H:quinone oxidoreductases (NQOs) (Table S1), of which 11 form complexes in our final network (Table S5). The components and functions of NQOs are not entirely consistent in different species [51]. The subcellular localizations of NQOs, which mediate electron transfer and shuttling of electrons from donors to quinones, are different in *E. coli*, *A. thaliana*, and *Synechocystis* [46], [47], [48]. NQOs are on the plasma membrane and participate in the respiratory process in *E. coli*, whereas the same complexes are found primarily on the thylakoid membrane in *A. thaliana* and *Synechocystis*, where they engage in the process of photosynthetic electron transport [49], [50]. We compared the PPIs of partial NQOs in *E. coli*, *A. thaliana*, and *Synechocystis.* We found that NdhM is only found in *Synechocystis*, while NdhJ is present in both *A. thaliana* and *Synechocystis*. In contrast, NdhK and NdhH are conserved in all three species (Figure 4B, left).

These results indicate that some components of the NQOs participate in the respiratory electron transport while others may have evolved new functions. For example, NdhJ and NdhM might be involved in photosynthesis in *A. thaliana* and *Synechocystis*. The appearance of homologs with roles in oxidation–reduction in *A. thaliana* can be a result of adapting to photosynthetic damage or participation in electron transport in *Synechocystis*. Reactive oxygen species (ROS) produced by aerobic metabolism in photoautotroph are disposed of by antioxidants [52], [53]. The presence of new enzymes with roles in oxidation–reduction can potentially reduce the influence of oxidative stress.

Bacterioferritin (Bfr) is an iron-storage protein, whose ferroxidase center binds to and oxidizes Fe^2+^ to Fe^3+^ by oxygen. The PPIs of Bfr proteins were studied and compared across three species. Intriguingly, no ortholog was found in *A. thaliana*, and only one ortholog (*i.e.*, Bfr) was found in *E. coli* (Figure 4B, right), suggesting that other proteins might perform this function in *A. thaliana.* Photoautotrophic microorganisms tend to have a massive demand for inorganic ions, such as Fe and Mn. Because inorganic ions are usually deficient in the open ocean, a unique mechanism for microbes to transport and store ions is required [54], [55]. The presence of two Bfr orthologs in *Synechocystis* indicates that it may have an additional requirement for iron compared to *E. coli* (Figure 4B). The protein components of Bfr complexes in *Synechocystis* have annotated functions linked to iron transport that are absent in *E. coli.* Like Bfr proteins and NQOs mentioned above, conservation analysis of macromolecules allows us to explore the biological processes within cells and how organisms adapt to different environmental demands.

### New roles for proteins related to cell mobility and lipid metabolism

**Figure 5:**
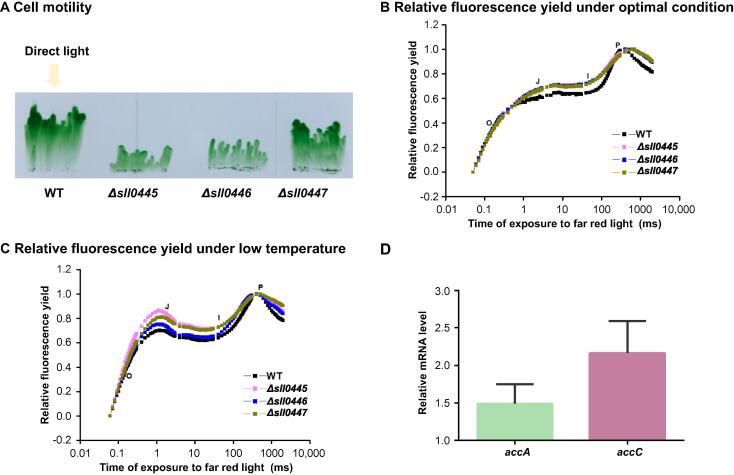
**Validation of protein new functions related to cell motility and lipid metabolism** **A.** The motility states of WT and mutants under unidirectional illumination (white light). The yellow arrow indicates the direction of the light source. **B.** and **C.** OJIP curve under the optimal condition or low temperature (20 °C). The J, I, and P steps occurred at about 2 ms, 30 ms, and 400 ms, respectively. O, origin (minimal fluorescence); P, peak (maximum fluorescence); J and I, inflection points between the O and P levels. **D.** qRT-PCR analysis showing the relative transcript levels of *accA* and *accC* (two genes encoding ACC subunits) in the *Δsll1334* mutant compared to those in the WT strain. Ct-values for each gene were normalized to that of *rnpB*. WT, wild-type; ACC, acetyl coenzyme A carboxylase.

As shown in Figure 3, hypothetical proteins Sll0445, Sll0446, and Sll0447 have putative interactions with pilus assembly proteins and photosystem complexes. Sll0445 contains a Tubulin_2 domain, and is a member of clan Tubulin (Figure S11A) which serves as cytoskeletal elements vital for cell division and material transport in all eukaryotes [56]. Sll0446 contains a FtsA domain, which is an actin-like ATPase domain, and co-localizes to the septal ring with FtsZ (Figure S11A) [57]. Sll0447 contains a DivIC domain, which is necessary for the formation of both vegetative and sporulation septa (Figure S11A) [58]. To assess these roles, we constructed interruption mutants for each of these three genes, with the insertion of a chloramphenicol-resistance cassette (Cm^R^) into their open reading frames (ORFs) (Figure S11B). Then the abilities of phototactic motility were tested and compared across the *Δsll0445*, *Δsll0446*, *Δsll0447*, and the wild-type (WT) strain. Under the unidirectional illumination, the WT stain showed obvious movement tendency toward the light, while the *Δsll0445*, *Δsll0446*, and *Δsll0447* mutants exhibited impaired phototaxis (Figure 5A). Moreover, a previous study showed that the expression level of *sll0447* was decreased in a mutant of *sycrp1* [42], a gene involved in phototactic movement [59], indicating that *sll0447* was regulated by Sycrp1 and influenced by cell motility.

The hypothetical proteins Sll0445, Sll0446, and Sll0447 not only formed protein complexes with pilus assembly proteins, but also interacted with photosystem complexes. Targeted removal of these genes from *Synechocystis* genome can affect electron transport of PSII under the optimal condition or low temperature (20 °C) (Figure 5B and C). *Synechocystis* exhibits a typical fluorescence induction polyphasic rise called the OJIP curve, which is similar to previous descriptions in plants, green algae, and cyanobacteria [60]. As shown in Figure 5B and C, the three mutants showed a sharp accumulation of fluorescence at phase J relative to the WT strain, suggesting that the reduction rate of Q_A_ and the oxidation rate of Q_A_^–^ were influenced by deletion of *sll0445*, *sll0446*, and *sll0447*.

While these results demonstrate that the proteins encode by *sll0445*, *sll0446*, and *sll0447* are necessary for the optimal function of photosystem and cell motility, it is still noteworthy that the pilus assembly proteins are distributed on the plasma membrane and the photosynthetic proteins are localized on the thylakoid membrane. Notably, part of the respiratory electron transport chain in *Synechocystis* is located in the thylakoid membrane and partially overlapped with the photosynthetic electron transport chain [49]. In our dataset, the PSI subunits form a complex with NADH dehydrogenase as part of the respiratory chain. The main purpose of *Synechocystis* motility is to obtain plenty of light, which consumes much energy during chemotaxis. Presumably, Sll0445, Sll0446, and Sll0447 link the structure of the thylakoid membrane with the plasma membrane to adjust the capture of light by the photosystem and chemotaxis.

As shown in Figure 3, the histidine kinase Sll1334 interacted with acetyl coenzyme A carboxylase (ACC). Sll1334 contains a GAF domain, which can sense and respond to light (Figure S11A) [61]. We observed that the transcript levels of the ACC subunits were up-regulated in the *sll1334* interruption mutant (*Δsll1334*) as compared with a WT parental strain (Figure 5D). The ACC complex catalyzes acetyl-CoA to form malonyl-CoA, which is part of lipid metabolism conserved across species [62]. Okada et al. have reported that *sll1334* may function as a suppressive regulator in this cascade, influencing cell growth and gene expression involved in glycometabolism under dark conditions [63]. The mechanism is still unclear as to how cyanobacteria adapt to dark conditions and use glucose as the carbon source for growth. However, our results may provide new insight into this process. Carbohydrate and lipid metabolisms drive central metabolism in living cells and have a close relationship with each other to control basic vital activities. Okada et al. found that *sll1330*, which encodes a histidine kinase, is located at the upstream of *sll1334* and influences the expression of *sll1334* [64]. Notably, *Δsll1330* and *Δsll1334* mutants did not grow well either under light activated heterotrophic or dark heterotrophic conditions as compared to the WT stain. However, they could grow as well as the WT stain under photoautotrophic conditions [63], [64]. These results indicate that carbohydrate and lipid metabolisms in *Synechocystis* are regulated by Sll1330 and Sll1334, and that light is an essential factor influencing this process.

Cyanobacteria can produce different bilin-binding photoreceptors when sensing various wavelengths of light, which adjust many essential cellular processes like growth, phototaxis, and photosynthesis to environmental light conditions [61]. The N-terminal region of photoreceptors was found as the photosensory module consisting of a ‘knotted’ structure including a GAF domain, a PAS domain, and a PHY domain, but not all proteins with a GAF domain are photoreceptors. Thus, further confirmation is needed to determine whether Sll1334 is a photoreceptor and how it regulates the ACC complex expression by sensing light.

### Integrative model derived from network systems biology

**Figure 6:**
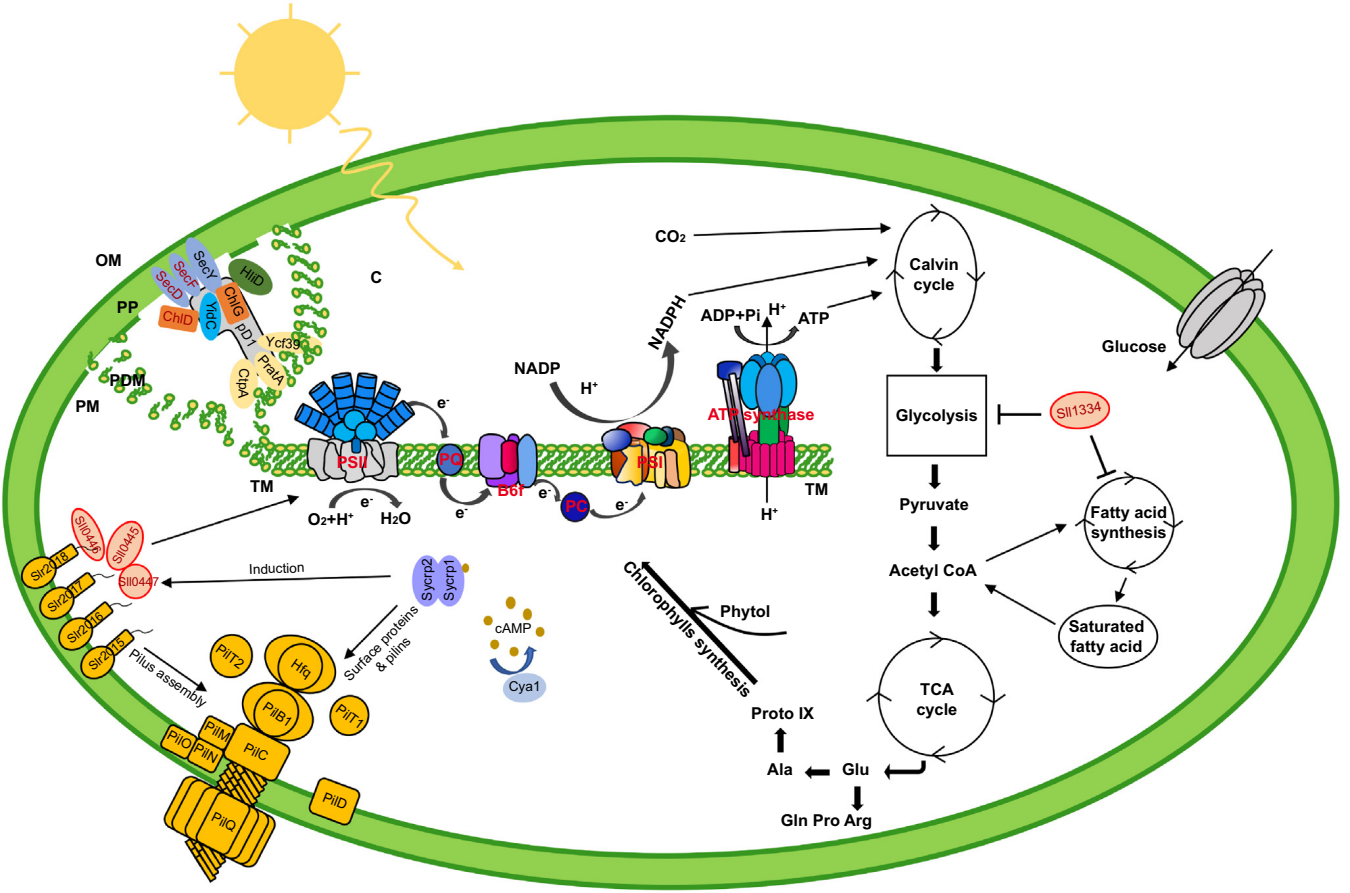
**The model of cyanobacterial phototaxis regulation** The model is based on our data (red color) and public knowledge, showing the phototaxis assembly and how it is influenced by the structure of pilus and carbon metabolism. Proteins SecD, SecF, and ChlD present a good elution profiling with PSII assembly proteins, indicating that Sec complexes and ChlD might play a critical role in the maturation of PSII. The hypothetical proteins Sll0445, Sll0446, and Sll0447 are induced by cAMP receptor protein Sycrp1 and interact with pilus assembly proteins to regulate cell motility. These hypothetical proteins can also interact with photosynthetic core proteins, revealing a close relationship between photosynthesis and cell motility in *Synechocystis*. Sll1334 can regulate glucolipid metabolism and may have an important role in controlling the utilization of sugars and lipids during heterotrophic growth, thereby affecting photosynthesis. pD1, precursor of D1; C, cytoplasm; L, lumen; OM, outer membrane; PDM, PratA-defined membrane; PM, plasma membrane; PP, periplasm; TM, thylakoid membrane.

Through integrating our data with the cyanobacterial metabolism pathway, we depicted a more comprehensive working model of phototaxis regulation, PSII assembly, and physiological metabolism in *Synechocystis* (Figure 6). Light is one of the essential elements to sustain life, especially for cyanobacteria and green plants that fix carbon from the outer environment by photosynthesis. To obtain enough light, cyanobacteria have been evolved with multiple abilities to adapt to light changes. For example, light energy can be harvested by large antenna complexes — phycobilisomes, while phototaxis allows *Synechocystis* to locate an ideal place to collect light [16], [65]. Phototaxis is influenced and regulated by light, the concentration of cAMP, and the structure of pilus in *Synechocystis* [16]. Slr2015–Slr2018 is one class of proteins involved in the process of pilus assembly and can be induced by cAMP receptor protein Sycrp1 [42]. The hypothetical proteins Sll0445, Sll0446, and Sll0447 are induced by Sycrp1 and interact with pilus assembly proteins, Slr2015, and Slr2018 to regulate cell motilities (Figure S8) [42]. Moreover, Sll0445, Sll0446, and Sll0447 influence photosynthesis through interactions with photosynthetic core proteins, revealing a close relationship between photosynthesis and cell motility in *Synechocystis* (Figure 5B and C).

We also provide more detailed information during the PSII assembly process by combining our predicted PPIs with previously published data (Figure 6). Biogenesis of PSII requires coordinated incorporation of at least 20 polypeptide subunits and a range of organic and inorganic cofactors [14]. Some of these components are well understood during the pratA-dependent PSII assembly process. For example, SecY, YidC, and CtpA facilitate D1 maturation from pD1; proteins ChlG, HliD, and Ycf39 are involved in the process of delivering the chlorophyll to new D1 or pD1 [34], [66], [67]. In addition, we observed SecD, SecF, and ChlD co-purifying with these proteins, indicating that Sec complexes and ChlD might play a critical function in the maturation of PSII during the pratA-dependent PSII assembly process (Figure 6).

*Synechocystis* can survive under photoautotrophic or heterotrophic growth conditions. The protein Sll1334 can regulate the expression of genes involved glucolipid metabolism. Its GAF domain may have an important role in controlling the utilization of sugars and lipids during heterotrophic growth.

Taken together, these proteins expand our understanding of the regulation of cell motility, PSII assembly, and glucolipid metabolism in *Synechocystis*.

## Conclusion

In this study, by combining CoFrac-MS and quantitative proteomics strategies, we predict 291 protein complexes consisting of 24,092 highly confident PPIs in *Synechocystis*, which is the largest PPI dataset for this species ever reported. This comprehensive PPI information greatly enhances the basic understanding of the molecular architecture and mechanisms of the photosynthesis machinery as well as other fundamental modules in cyanobacteria.

From the predicted PPIs, most of the proteins tend to have lower degrees involved in metabolic regulation, whereas the proteins with higher degrees are endowed with more basic and conserved functions in *Synechocystis*. By separating photosynthetic protein interaction networks from whole PPIs, we elucidate how photosynthetic proteins connect with other functional units and influence disparate biological processes. By comparing protein complexes in *Synechocystis* with other species, including *A. thaliana*, *E. coli*, *H. sapiens*, and *S. cerevisiae*, we observe macromolecular evolution and functional variations in different species. For example, the change of NQO components in different species may reflect that photosynthetic organisms, like *Synechocystis* and *A. thaliana*, have to undertake the process of photosynthesis and recovery from photosynthetic damage. According to the predicted complexes, the hypothetical proteins Sll0445, Sll0446, and Sll0447 were found to build a functional connection between photosynthesis and cell motility. The photosynthetic apparatus serves as a regulator in the energy metabolic process in living cells. Photosynthesis has a close relationship with chemotaxis, because one of the primary purposes of cell motility is to maximize light exposure so that photosynthesis can tap light energy to fix CO_2_ to provide energy for cellular processes. Moreover, expression of the ACC complex was up-regulated when the new component Sll1334 was depleted. Cyanobacteria are considered as a promising organism for producing biofuels, but current productivity still needs further improvement. Since Sll1334 was found to be a negative regulator of glycolipid metabolism, our work suggests new avenues to improve biofuel productivities by genetic modification.

In summary, the global landscape of native protein complexes in *Synechocystis* provides a valuable resource for researchers to find and determine new and promising macromolecules for further investigations. It also expands our knowledge of the functional interaction network that governs the molecular biology of cyanobacteria.

## Materials and methods

### Growth condition and protein extraction

*Synechocystis* strain was grown in liquid BG11 medium at 30 °C in the light (30 μmol·m^−2^·s^−1^). The cells were collected by centrifugation (6000 *g* at 4 °C for 5 min) when grew to the exponential phase (OD_730_ = 0.8–1). The cells were lysed with lysis buffer containing 20 mM Tris-HCl (pH 7.5), 150 mM NaCl, 1% DDM (Catalog No. D4641, Merck, Darmstadt, Germany), and Complete Protease Inhibitors EDTA-free (Catalog No. 4693124001, Roche, Basel, Switzerland), and then sonicated (5 s on, 10 s off) for about 5 min on ice with an output of 135 W. The cell debris was discarded by centrifugation (12,000 *g* at 4 °C for 10 min). The protein concentration of each sample was measured using the Bradford assay.

### SEC, IEX, and SDGC

*Synechocystis* cell lysates were fractionated by SEC and IEX on an Ultimate 3000 HPLC system (ThermoFisher Scientific, Bremen, Germany). For SEC, the lysates were injected (350 μl per injection) onto MAbPac SEC-1 (5 μm, 300 mm × 4.0 mm; ThermoFisher Scientific) or Superose 6 10/300GL column (GE Life Sciences). There were 24 fractions collected by using MAbPac SEC-1, with a flow rate of 0.2 ml/min, and 45 fractions collected by using Superose 6 10/300GL column, with a flow rate of 0.3 ml/min. Protein standards (thyroglobulin, BSA, Albumin egg, and myoglobin) were analyzed with the same method to obtain the approximate MW range across fractions. For IEX, the ion-exchange column (12 μm, 200 mm × 4.6 mm, 1500 Å; Columnex, San Diego, CA) was used, and a 110 min salt gradient (0.12–1.2 M NaCl) was used to collect 43 fractions. The elution buffer A containing 10 mM Tris-HCl (pH 7.6), 0.5 mM DTT, and 5% glycerin, while elution buffer B with additional 1.2 M NaCl. For SDGC, lysates were loaded onto a 12 ml 15%–70% (w/v) linear sucrose gradient, which were then centrifuged at 160,000 *g* at 4 °C for 16 h in a Beckman MLS-50 rotor (Beckman-Coulter, CA), and 24 fractions were collected. In total, 181 fractions were collected.

### APMS

The target protein was combined with a green fluorescent protein (GFP) at its C-terminus. Cell lysate containing the GFP-tagged protein was subjected to affinity purification by using Anti-GFP antibody (Catalog No. ab290, Abcam, Cambridgeshire, UK). The process of antibody purification was carried out using GenScript Protein A MagBeads (Catalog No. L00273, GenScript, Piscataway, NJ) according to the manufacturer’s instructions. Then, the sample was detected by MS.

### Trypsin digestion and peptide clean up

Proteins from all HPLC fractions were precipitated with 10% trichloroacetic acid at 4 °C overnight and dissolved in 50 mM ammonium bicarbonate. Trypsin (Catalog No. V5113, Promega, Madison, WI) was added at the ratio of 1:50 and incubated overnight at 37 °C. Each fraction was desalted using ZipTip C18 plates (Catalog No. ZTC18S960, Millipore, Darmstadt, Germany). Peptides were dried using a Labconco evaporator and then resuspended in 0.1% formic acid for further analyses.

### Nano-LC-MS/MS analysis

The peptides were dissolved in 0.1% formic acid, and analyzed using Q-Exactive Plus Orbitrap mass spectrometer (ThermoFisher Scientific). Peptides in 0.1% formic acid were separated on a C18 nano-trap column at a flow rate of 500 nl/min. Peptides were ionized at 2.0 kV. The precursor ions were fragmented by using high energy collision induced dissociation (HCD). The MS/MS spectra of the top 20 most intense signals were acquired by using a data-dependent method. The dynamic exclusion duration was set as 40 s and 5 × 10^4^ ions were set to generate MS/MS spectra in the automatic gain control (AGC). The Proteome Discoverer version 2.1 was used to retrieve the RAW data using a target-decoy based strategy, supplied with the *Synechocystis* 3508 reference protein (UP000001425) from the UniProt database. Up to 2 missed cleavages were allowed.

### Data analysis

R Language and Python scripts were applied to data analysis. The elution profiles for individual proteins were normalized and smoothed by using scale command in R Language. The Mapp of all proteins identified in our dataset was calculated similarly as previously described [26]. After that, the ratio of Mapp to Mmono was calculated, and the value of Rapp (Mapp/Mmono) can effectively reflect the oligomerization state of proteins during the protein separation process. The protein with a value of Rapp ≥ 2 implies an oligomerization state, while Rapp ≤ 0.5 means that it may be degraded during the protein extraction process and would be discarded in subsequent analysis. The protein with a value of 0.5 < Rapp < 2 exits as a monomeric state.

### Machine learning

We used EPIC software for automated scoring of our data for the large-scale determination of high-confidence physical interaction networks and macromolecular assemblies from diverse biological specimens. This software package can be obtained from https://github.com/BaderLab/EPIC. Protein pairs were scored based on five features: MI, Bayes Correlation, Jaccard, Pearson Correlation Coefficient, and Apex Score. We manually collected a data set of “gold standard” protein complexes by the reference database (UniProt) for machine learning analysis, which contains 48 conserved true positive protein complexes. Positive PPIs are defined if they appear in the same protein complex, while the components of negative PPIs are from proteins existing in the different protein complexes. Then the positive and negative PPIs were used to train the machine learning classifier. The protein pairs with elution profile similarity scores more than 0.5 were required, and the proteins that used for machine learning were detected with not less than 2 peptide spectrum matches in at least one of the experiments.

### Construction of plasmids

Single mutants of the *sll0445*–*sll0447* gene cluster and *sll1334* were generated by inserting a Cm^R^ into their ORFs. For APMS, GFP-tag was added to the C-termini of *sll0445* and *slr0149* in the genome. The targeted gene and its flanking sequences were amplified by PCR with *Synechocystis* chromosome DNA as the template and cloned into the pMD18–T vector (Catalog No. D101A, Takara, Japan). The insertion mutants were verified by PCR (Figure S11B). Primers used for mutant construction are as follows: M_*sll0445*up, 5′-GTTCAGCGGTGATGAGTVG-3′; M_*sll0445*down, 5′-GTAAATCAAACAGGGCATG-3′; M_*sll0446*up, 5′-TGTGGCCTATACAATGTCCCAG-3′; M_*sll0446*down, 5′-AAGATATTTCTTCCAGCAAATGG-3′; M_*sll0447*up, 5′-ATCTCGTATTAAGAAAGCTTG-3′; M_*sll0447*down, 5′-TGAGCATAAACTGGACTAATG-3′; M_*sll1334*up, 5′-AGACGGTTAGAACCAACAGTCACTG-3′; M_*sll1334*down, 5′-ACAATTTGTAAGCCCTGGCGAACG-3′.

### Cell motility assay and measurement of photosynthetic activity

Phototactic movement was tested according to Wilde and colleagues [68]. The strains were grown on solid BG11 medium containing 0.3% sodium thiosulfate, 8 mM TES (pH 8.0), 0.8% agar, and 5 mM glucose, under unidirectional illumination with light intensity at 1–5 μmol photons m^−2^·s^−1^ and the movements were recorded at day 6. The modification of PSII photochemistry in *Synechocystis* was evaluated by OJIP curve, measured by Plant Efficiency Analyzer (Hansatech, Germany).

### RNA isolation and qRT-PCR analysis

About 50 ml of *Synechocystis* grown in BG11 was collected by centrifugation at 4 °C and the total RNA was extracted using the TRIzol Reagent (Catalog No. 15596-026, Invitrogen, Waltham, MA). The cDNA was synthesized with the Perfect Real Time Kit (Catalog No. RR047A, Takara, Japan), and used as a template for qRT-PCR analysis. RNase P subunit B (*rnpB*) was used as an internal control. Primers used for qRT-PCR are as follows: *accA*up, 5′-AAATGTTTCGGTTAGATGTCC-3′; *accA*down, 5′-CCAAAGAATAGCCGCACA-3′; *accC*up, 5′-TTTGGTGGATGGTAACGG-3′; *accC*down, 5′-TGGCGGAAGCGGAGTTTT-3′; *rnpB*up, 5′-ACCGCTTGAGGAATTTGGTA-3′; *rnpB*down, 5′-TTAGTCGTAAGCCGGGTTCT-3′.

## Data availability

All LC-MS/MS raw data related to this work have been deposited to the ProteomeXchange Consortium via the iProX partner repository (ProteomeXchange: PXD015948; iProX: IPX0001620001), which are publicly accessible at http://proteomecentral.proteomexchange.org and https://www.iprox.org, respectively.

## CRediT author statement

**Chen Xu:** Investigation, Visualization, Writing - original draft. **Bing Wang:** Resources. **Lin Yang:** Visualization. **Lucas Zhongming Hu:** Software. **Lanxing Yi:** Investigation. **Yaxuan Wang:** Investigation. **Shenglan Chen:** Investigation. **Andrew Emili:** Writing - review & editing. **Cuihong Wan:** Conceptualization, Supervision, Writing - review & editing. All authors have read and approved the final manuscript.

## Competing interests

The authors declare no competing interests to this work. We declare that we do not have any commercial or associative interest that represents a conflict of interest in connection with the work submitted.

## Supplementary material

Supplementary Figure S1 **The complementarity of protein elution profiling in SEC and IEX** The proteins that are not separated effectively in SEC (left) are protein complexes with MW beyond the SEC valid separation range and are eluted in early fractions. However, some of these protein complexes can be separated by IEX according to their elution profiling (right). Red color corresponds to protein abundance.

Supplementary Figure S2 **The distribution of average MW or pI in all fractions A.** MAbPac SEC-1 column. **B.** Superose 6 10/300GL column. **C.** IEX mixed-bed ion-exchange column. For each fraction, the average MW or pI is average MW or pI of all proteins identified in that fraction. The dash lines are trending line.

Supplementary Figure S3 **Regression curve of standard proteins** Protein standards of known MW (thyroglobulin, BSA, Albumin egg, and myoglobin) were separated by SEC column, and their elution peaks were used to calculate approximate MW of the fractions. **A.** MAbPac SEC-1 column and **B.** Superose 6 10/300GL column.

Supplementary Figure S4 **Elution profiles of components of known protein complexes** Elution profiling of Photosystem I, RubisCO, C-phycocyanin, NAD(P)H-quinone oxidoreductase, and Triosephosphate isomerase on three different columns (SEC1: MAbPac SEC, SEC2: Superose 6 10/300GL, IEX mixed-bed ion exchange). The elution profiling lines of proteins in one protein complex are shown in different colors. x-axis: elution fraction number, y-axis: normalized label-free quantification intensity.

Supplementary Figure S5 **Evaluation results for machine learning A.** Precision/Recall vs correlation score from experimental data. The plot shows the classifier performance in predicting co-complex membership. **B.** Precision-recall curve (PR) for co-complex PPI prediction from experimental data.

Supplementary Figure S6 **The distributions of proteins degrees in different organisms** The protein-protein interaction pairs of *Synechocystis* were generated from our dataset, and PPIs of other model organisms were obtained from the Mentha database. The degree is defined as the number of edges that one protein links to other proteins in the network.

Supplementary Figure S7 **Organization of the three CRISPR/Cas systems in *Synechocystis*** The CRISPR/Cas systems were illustrated based on previous work [37,38]. The Cas-genes are represented by arrows located upstream of the CRISPR arrays. Arrows in white represent proteins not identified by MS. Arrows in gray illustrate those proteins, for which no high confident physical interactions were found in our dataset. Other arrows in the same colors represent those proteins that can form complex, such as the CRISPR3 can form one complex (red color), and CRISPR2 can interact with both CRISPR1 (green) and CRISPR3 (purple).

Supplementary Figure S8 **The APMS results of Slr0149** Schematic illustration of the PPIs involving protein Slr0149 from Co-fractionation and APMS data. The mass spectrometry Peptide-Spectrum Match (PSM) numbers of proteins co-purified with Slr0149 from GFP-tagged APMS listed in Table S6.

Supplementary Figure S9 **Conservative analysis of predicted complexes** The proportion of different similarity degrees of all *Synechocystis* protein complexes in this work was plotted. The similarity degree was calculated by the percentage of protein components in each complex that has homologous in other organisms.

Supplementary Figure S10 **Comparative analysis of proteins annotated with oxidation-reduction** Bubble graphs demonstrate the gene ontology terms (biological process) (x-axis) plotted against the –log10*P* value for oxidation-reduction related protein with homologs in *A. thaliana* part (**A**) and homologs in *A. thaliana* and *E. coli* part (**B**), respectively.

Supplementary Figure S11 **Construction and detection of the *Synechocystis sll0445–sll0447* gene cluster and s*ll1334* A.** The predicted domains of proteins Sll0445–Sll0447 (Top) and Sll1334 (Bottom). *sll0445* encodes a protein with a Tubulin_2 domain, *sll0446* encodes a protein with a FtsA domain, and *sll0447* encodes a protein with a DivIC domain. *sll1334* encodes a protein with GAF and HisKA domains. **B.** Detection of the degree of segregation of the *sll0445–sll0447* mutants by PCR amplification. Lanes 1–2 use primers *sll0445* up and *sll0445* down. Lanes 3–4 use primers *sll0446* up and *sll0446* down. Lanes 5–6 use primers *sll0447* up and *sll0447* down. Lanes 7–8 use primers *sll1334* up and *sll1334* down. Lanes 1, 3, 5, and 7: wild type strain DNA; lane 2: *sll0445*::CmR mutant DNA; lane 4: *sll0446*::CmR mutant DNA; lane 6: *sll0447*::CmR mutant DNA; lane 8: *sll1334*::CmR mutant DNA.

Supplementary Table S1 LC/MS/MS identification and quantification of all fractionations.

Supplementary Table S2 List of gold standard complexes for machine learning.

Supplementary Table S3 Output PPIs from EPIC toolkit.

Supplementary Table S4 Description and GO annotation of photosynthesis associated proteins.

Supplementary Table S5 Final 291 predicted protein complexes of *Synechocystis* sp. PCC 6803.

Supplementary Table S6 Affinity purification mass spectrometry results.

Supplementary Table S7 Protein with homologous in different species.
