# Supplementary figures and images for: Global Landscape of Native Protein Complexes in Synechocystis sp. PCC 6803
Source: Genomics Proteomics Bioinformatics. 2021 Feb 24;20(4):715–27. doi: 10.1016/j.gpb.2020.06.020 (PMC9880817; doi:10.1016/j.gpb.2020.06.020)

# SEC

Fractions

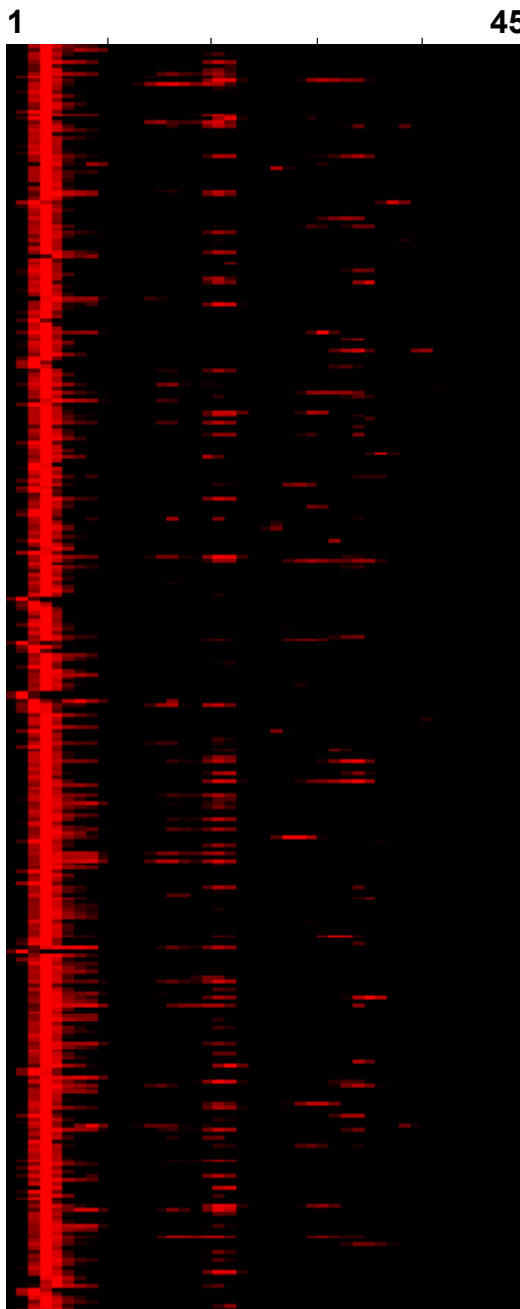

# PolyCATWAX

Fractions

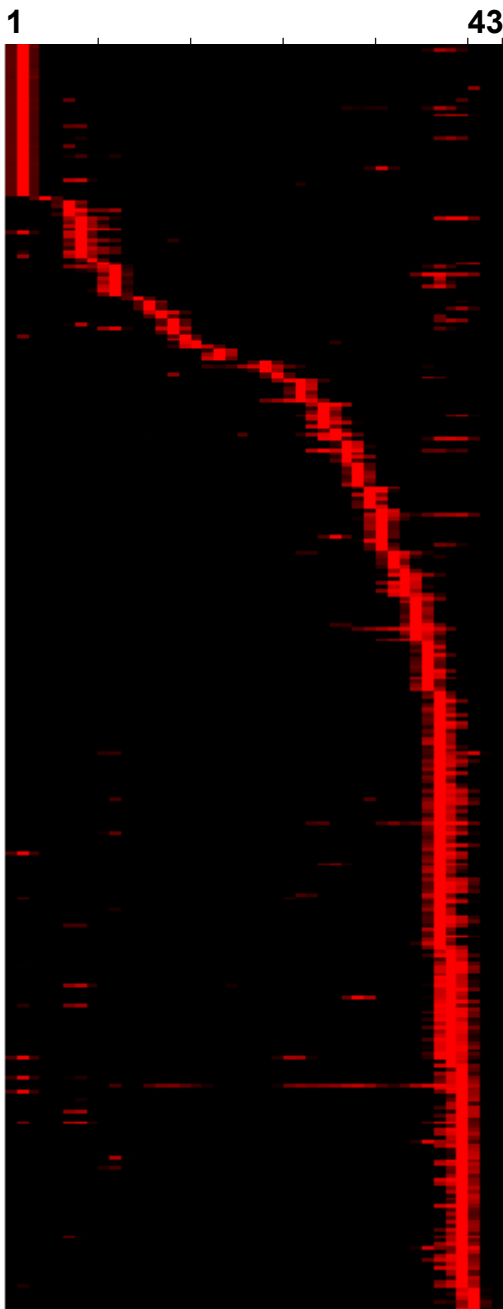

Normalized  
Intensity

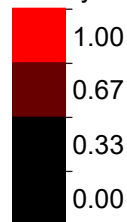

Identified protein

Supplement: Supplementary Figure S1 — The complementarity of protein elution profiling in SEC and IEX The proteins that are not separated effectively in SEC (left) are protein complexes with MW beyond the SEC valid separation range and are eluted in early fractions. However, some of these protein complexes can be separated by IEX according to their elution profiling (right). Red color corresponds to protein abundance. [file mmc1.pdf]

A Regression curve of standard proteins in SEC1

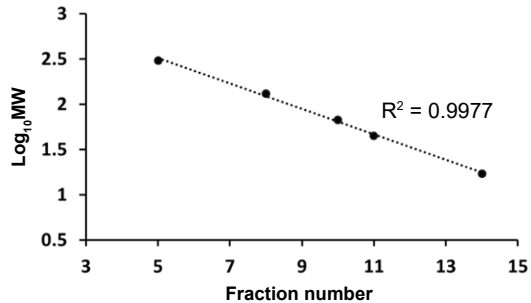

B Regression curve of standard proteins in SEC2

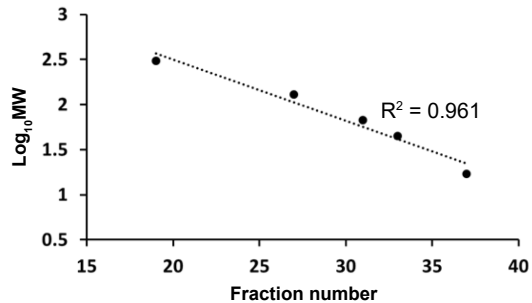

Supplement: Supplementary Figure S3 — Regression curve of standard proteins Protein standards of known MW (thyroglobulin, BSA, Albumin egg, and myoglobin) were separated by SEC column, and their elution peaks were used to calculate approximate MW of the fractions. A. MAbPac SEC-1 column and B. Superose 6 10/300GL column. [file mmc3.pdf]

SEC1

SEC2

PolyCATWAX

Normalized LFQ intensity

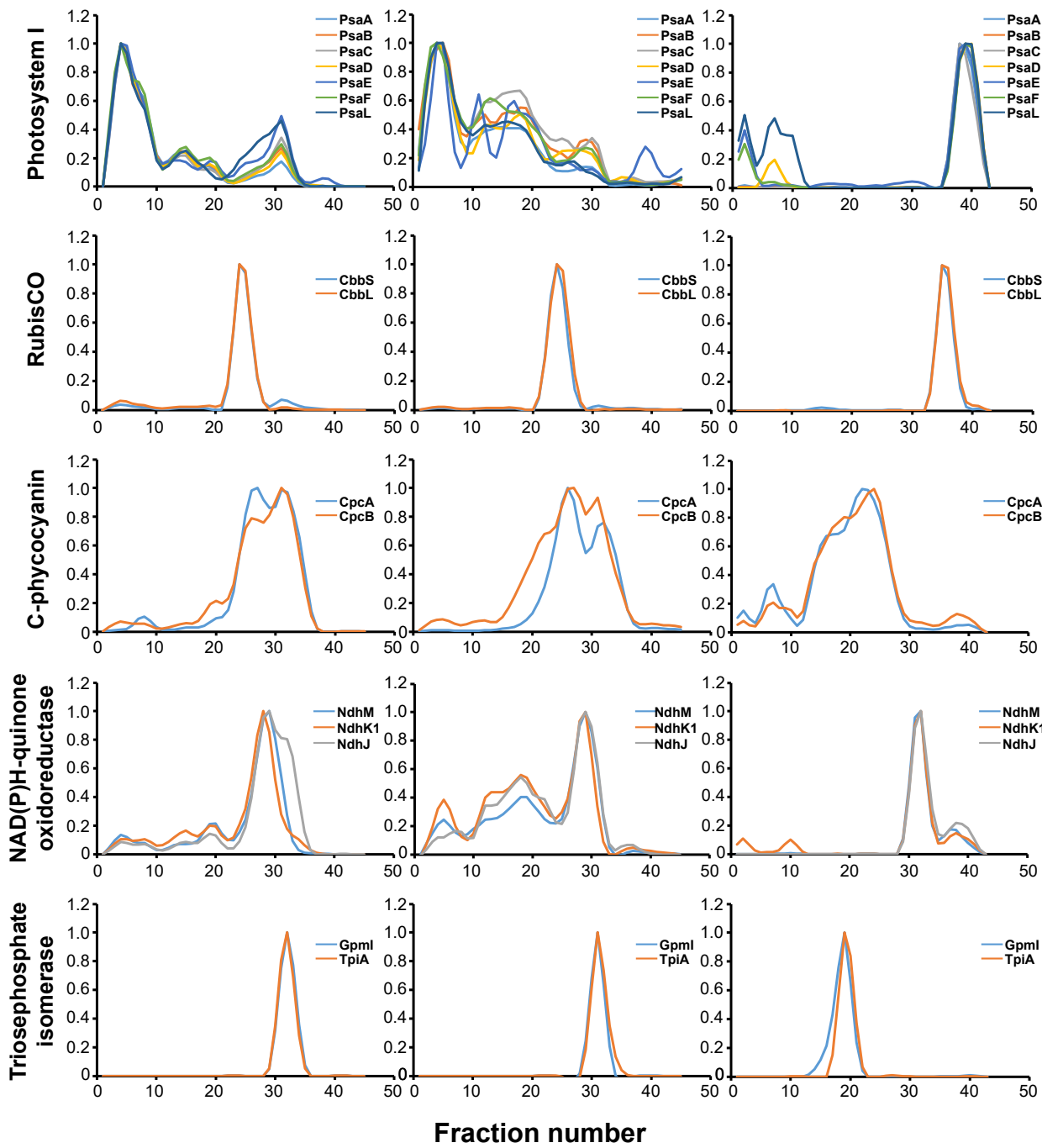

Supplement: Supplementary Figure S4 — Elution profiles of components of known protein complexes Elution profiling of Photosystem I, RubisCO, C-phycocyanin, NAD(P)H-quinone oxidoreductase, and Triosephosphate isomerase on three different columns (SEC1: MAbPac SEC, SEC2: Superose 6 10/300GL, IEX mixed-bed ion exchange). The elution profiling lines of proteins in one protein complex are shown in different colors. x-axis: elution fraction number, y-axis: normalized label-free quantification intensity. [file mmc4.pdf]

**A**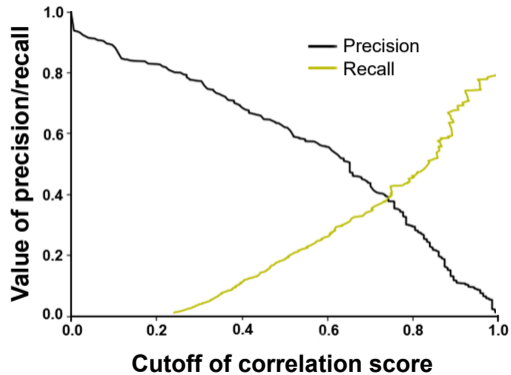**B**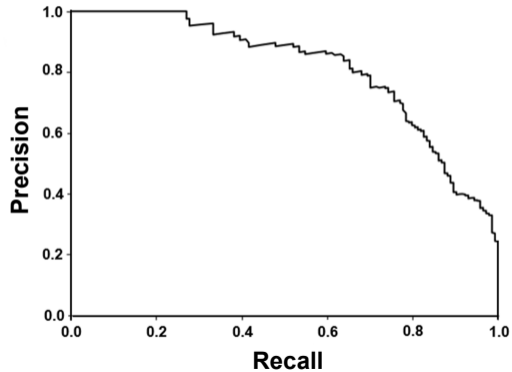

Supplement: Supplementary Figure S5 — Evaluation results for machine learning A. Precision/Recall vs correlation score from experimental data. The plot shows the classifier performance in predicting co-complex membership. B. Precision-recall curve (PR) for co-complex PPI prediction from experimental data. [file mmc5.pdf]

The proportion of proteins with  
different degrees

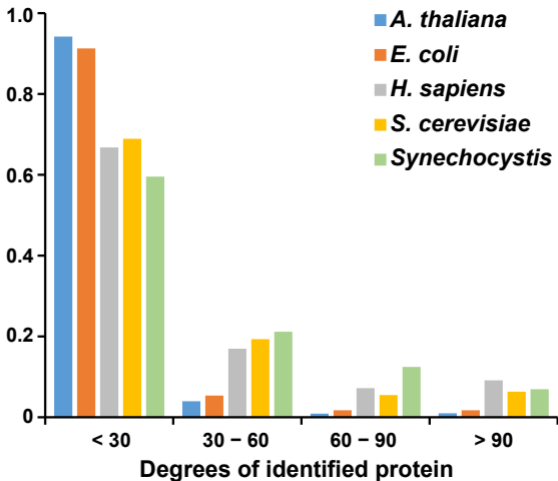

Supplement: Supplementary Figure S6 — The distributions of proteins degrees in different organisms The protein-protein interaction pairs of Synechocystis were generated from our dataset, and PPIs of other model organisms were obtained from the Mentha database. The degree is defined as the number of edges that one protein links to other proteins in the network. [file mmc6.pdf]

## CRISPR 1

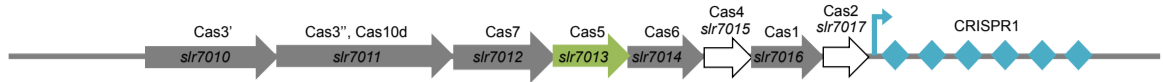

## CRISPR 2

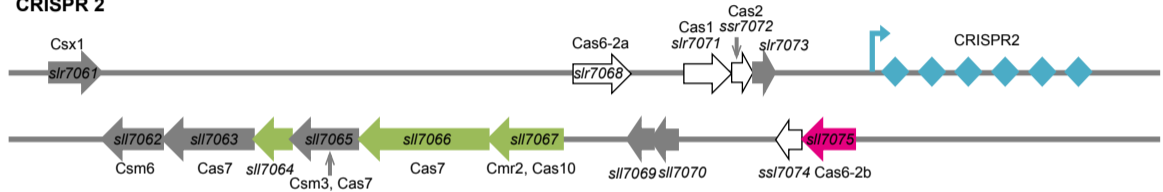

## CRISPR 3

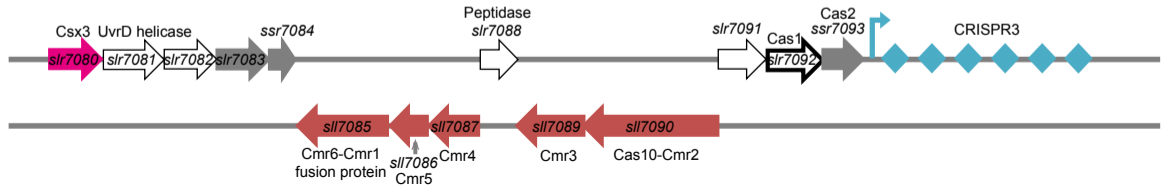

Supplement: Supplementary Figure S7 — Organization of the three CRISPR/Cas systems in Synechocystis The CRISPR/Cas systems were illustrated based on previous work [37,38]. The Cas-genes are represented by arrows located upstream of the CRISPR arrays. Arrows in white represent proteins not identified by MS. Arrows in gray illustrate those proteins, for which no high confident physical interactions were found in our dataset. Other arrows in the same colors represent those proteins that can form complex, such as the CRISPR3 can form one complex (red color), and CRISPR2 can interact with both CRISPR1 (green) and CRISPR3 (purple). [file mmc7.pdf]

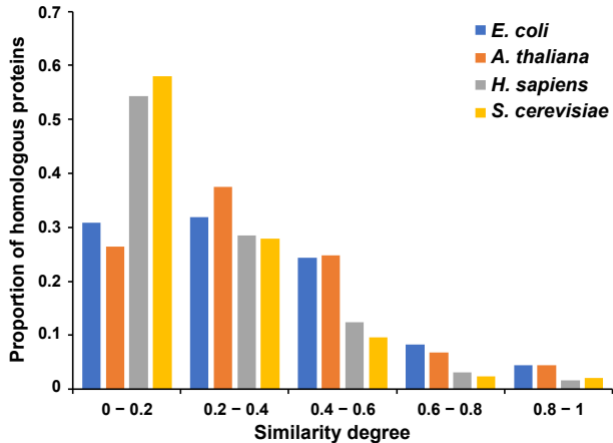

Supplement: Supplementary Figure S9 — Conservative analysis of predicted complexes The proportion of different similarity degrees of all Synechocystis protein complexes in this work was plotted. The similarity degree was calculated by the percentage of protein components in each complex that has homologous in other organisms. [file mmc9.pdf]

## A Homologous with *A. thaliana* part

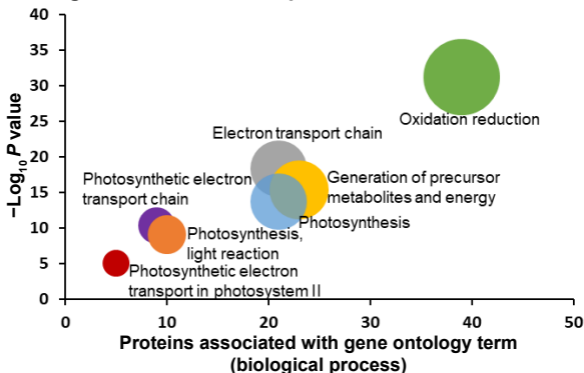

## B Homologous with *A. thaliana* and *E. coli* part

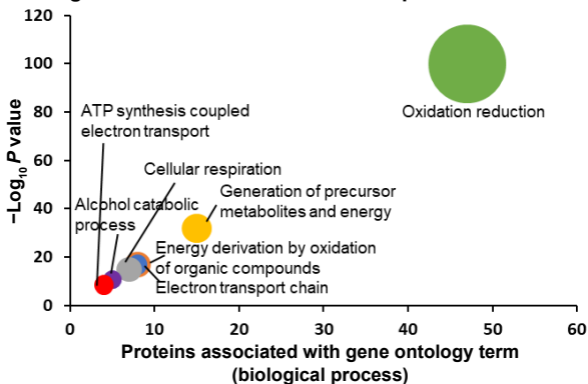

Supplement: Supplementary Figure S10 — Comparative analysis of proteins annotated with oxidation-reduction Bubble graphs demonstrate the gene ontology terms (biological process) (x-axis) plotted against the –log10P value for oxidation-reduction related protein with homologs in A. thaliana part (A) and homologs in A. thaliana and E. coli part (B), respectively. [file mmc10.pdf]
