# Supplementary material for: Global Landscape of Native Protein Complexes in Synechocystis sp. PCC 6803
Source: Genomics Proteomics Bioinformatics. 2021 Feb 24;20(4):715–27. doi: 10.1016/j.gpb.2020.06.020 (PMC9880817; doi:10.1016/j.gpb.2020.06.020)

A The distribution of average MW in SEC1

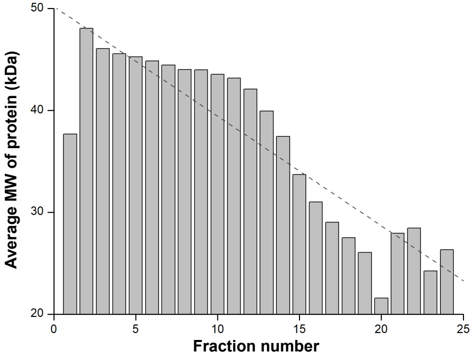


B The distribution of average MW in SEC2

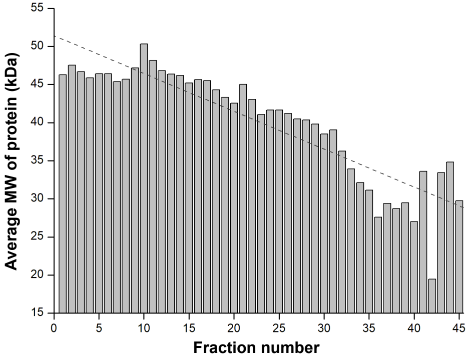


C The distribution of average pI in IEX

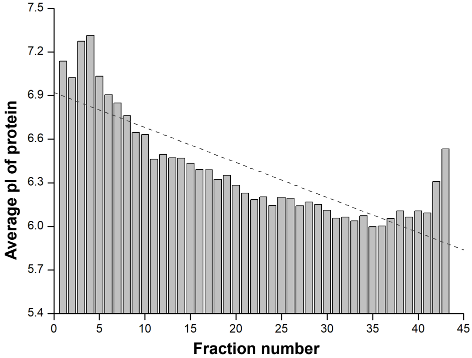

Supplement: Supplementary Figure S2 — The distribution of average MW or pI in all fractions A. MAbPac SEC-1 column. B. Superose 6 10/300GL column. C. IEX mixed-bed ion-exchange column. For each fraction, the average MW or pI is average MW or pI of all proteins identified in that fraction. The dash lines are trending line. [file mmc2.pdf]
