# Supplementary material for: Global Landscape of Native Protein Complexes in Synechocystis sp. PCC 6803
Source: Genomics Proteomics Bioinformatics. 2021 Feb 24;20(4):715–27. doi: 10.1016/j.gpb.2020.06.020 (PMC9880817; doi:10.1016/j.gpb.2020.06.020)
Supplement: Supplementary Figure S8 — The APMS results of Slr0149 Schematic illustration of the PPIs involving protein Slr0149 from Co-fractionation and APMS data. The mass spectrometry Peptide-Spectrum Match (PSM) numbers of proteins co-purified with Slr0149 from GFP-tagged APMS listed in Table S6. [file mmc8.pdf]

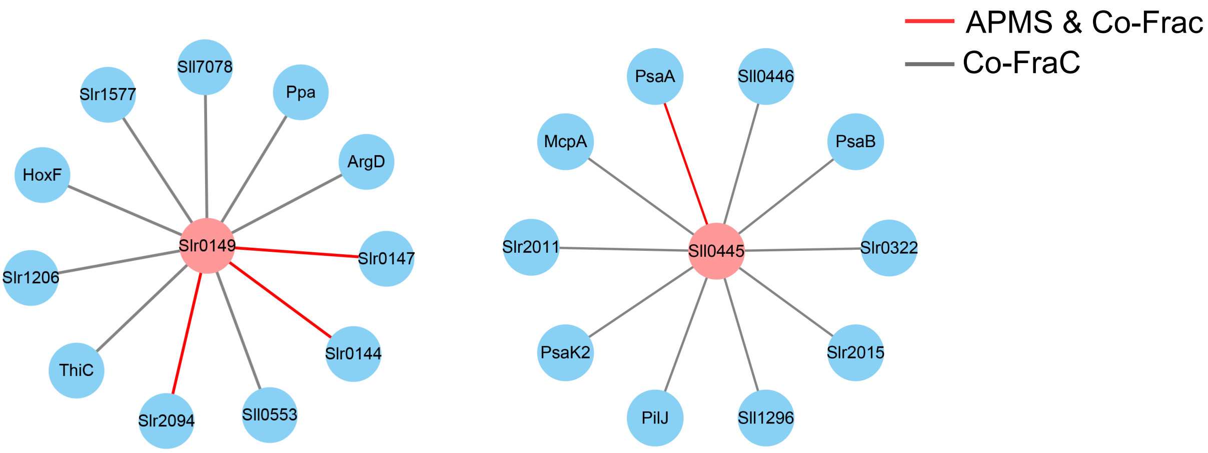

	Protein	PSM count
**Bait**	**Slr0149-GFP**	**12**
Prey	Slr0144	6
	Slr0147	6
	Slr2094	3
**Bait**	**Sll0445-GFP**	**14**
Prey	PsaA	5
	PsaF	8
	Slr2018	8
