# Supplementary material for: Global Landscape of Native Protein Complexes in Synechocystis sp. PCC 6803
Source: Genomics Proteomics Bioinformatics. 2021 Feb 24;20(4):715–27. doi: 10.1016/j.gpb.2020.06.020 (PMC9880817; doi:10.1016/j.gpb.2020.06.020)
Supplement: Supplementary Figure S11 — Construction and detection of the Synechocystis sll0445–sll0447 gene cluster and sll1334 A. The predicted domains of proteins Sll0445–Sll0447 (Top) and Sll1334 (Bottom). sll0445 encodes a protein with a Tubulin_2 domain, sll0446 encodes a protein with a FtsA domain, and sll0447 encodes a protein with a DivIC domain. sll1334 encodes a protein with GAF and HisKA domains. B. Detection of the degree of segregation of the sll0445–sll0447 mutants by PCR amplification. Lanes 1–2 use primers sll0445 up and sll0445 down. Lanes 3–4 use primers sll0446 up and sll0446 down. Lanes 5–6 use primers sll0447 up and sll0447 down. Lanes 7–8 use primers sll1334 up and sll1334 down. Lanes 1, 3, 5, and 7: wild type strain DNA; lane 2: sll0445::CmR mutant DNA; lane 4: sll0446::CmR mutant DNA; lane 6: sll0447::CmR mutant DNA; lane 8: sll1334::CmR mutant DNA. [file mmc11.pdf]

A

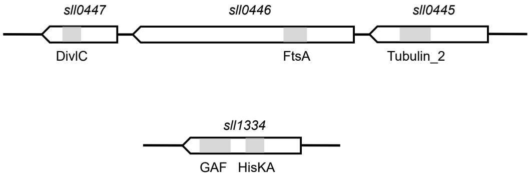

sll0447
sll0446
sll0445
DivIC
FtsA
Tubulin_2
sll1334
GAF
HisKA

B

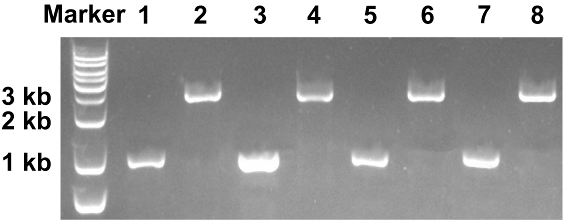

Marker
1
2
3
4
5
6
7
8
3 kb
2 kb
1 kb
